# Online information on bowel resection for Crohn’s disease

**DOI:** 10.1308/rcsann.2025.0108

**Published:** 2026-01-12

**Authors:** A Whitman, N Husnoo, J Johnston, L Wyld, S Brown

**Affiliations:** ^1^ University of Hull, UK; ^2^ Hull York Medical School, UK; ^3^ University of Sheffield, UK; ^4^ Sheffield Teaching Hospitals NHS Foundation Trust, UK; ^5^ Bradford Teaching Hospitals NHS Foundation Trust, UK; ^6^ Doncaster and Bassetlaw Teaching Hospitals NHS Foundation Trust, UK

**Keywords:** Crohn’s disease, Shared decision making, Bowel resection, Patient empowerment.

## Abstract

**Introduction:**

Most patients with Crohn’s disease (CD) have at least one bowel resection during their lifetime. Patients considering surgery will probably look for information online, as is common practice among patients with chronic illnesses. The aim of this systematic review is to assess the quality and readability of web-based patient information on bowel resection for CD.

**Methods:**

Google was searched using predefined search terms, developed with input from patient experts. For each term, results from the first two pages were screened for eligibility. Patient-focused websites on bowel resection for CD were included. The quality of the information was assessed using the DISCERN tool, and the readability with the Flesch–Kincaid ease of readability (FK) score. The accessibility adjustments of websites were also assessed.

**Results:**

Of the 118 sources identified, 91 were excluded and 27 sources were analysed. One-third (*n* = 10) did not discuss the different types of resections. Ileocolic resection (the most commonly performed resection) was described in eight sources. Discussion of management post-resection (*n* = 6) and of lifestyle changes (*n* = 11) was sparse. There were some instances of factually incorrect information. The mean DISCERN score was 3.1 ± 0.80 (range 1–5), indicating moderate quality information. The mean FK score was 51.9 ± 8.70 (corresponding to patients requiring A levels or equivalent to fully understand the text).

**Conclusions:**

The study findings highlighted the limitations of the current online patient information surrounding bowel resection in CD. The involvement of patients, working alongside professional bodies and clinicians, in the development of health-related websites is recommended.

## Introduction

Crohn’s disease (CD) is a subtype of inflammatory bowel disease (IBD) that is most prevalent in North America and Western Europe.^1,2^ There is currently no cure for CD but active disease can be managed by medical therapy, surgery or a combination of both.^3^

Significant advances have occurred in the medical treatment of CD in the past 25 years (since biologics entered clinical practice in the late 1990s). Despite this, most patients require a bowel resection for CD at some time during the course of their disease, with at least half needing surgery within ten years of diagnosis.^4^ Currently, the conventional first-line treatment for patients diagnosed with CD is medical therapy, often with steroids to induce remission, followed by immunosuppressants and/or biologics to maintain remission.^5^ Surgery has traditionally been considered an option when disease complications (such as stricturing or fistulisation) arise, or when medical therapy has not worked. However, recent studies have highlighted the benefits of earlier bowel resection as an alternative to medical therapy, especially in isolated terminal ileal disease (the site that is most commonly affected by CD), in reducing the risk of recurrence and the need for medication and subsequent surgery.^6–10^ Surgery therefore continues to have a significant role in the management of CD.

Having major abdominal surgery, for a chronic condition with potential to recur, is likely to represent a significant event for a patient. It is recognised that patients want to be involved in decisions about their care, hence the shift towards shared decision making (SDM) in recent years^11,12^ to ensure that patients are involved in the decision making about their care if they wish.^13^ The key to SDM is the patient’s access to good-quality, evidence-based information.^13^ The patient with CD facing a bowel resection should have sufficient understanding of what the procedure involves to enable them to make an informed decision.^14,15^

The internet is increasingly being used by the public as a source of health-related information.^16^ Previous studies have demonstrated that accessing online information can improve health literacy and empower patients to feel more in control of their disease.^17,18^ Although gastroenterologists are often the main source of information about treatment, most IBD patients use the internet as an additional source, especially about surgery.^19^ Therefore, it is vital to have accurate and trustworthy online patient-focused information surrounding bowel resection in CD, to complement the information received from clinicians.

The aim of this systematic review was to assess the quality and readability of web-based patient information on bowel resection in CD.

## Methods

The protocol for this review was registered on Open Science Framework (OSF) (https://doi.org/10.17605/OSF.IO/CEZ9D). The study is reported in accordance with Preferred Reporting Items for Systematic Reviews and Meta-analyses (PRISMA) guidelines.^20^

### Search strategy

A search was carried out on the Google Search engine between 27 November and 4 December 2023, using six different search terms one at a time. Google was chosen because it is the most used search engine in the world.^21^ Search terms were proposed by patient experts who have experience of previous bowel resections for CD, identified from a ‘patient and public involvement’ group involved in CD-related research. The terms were refined by the research team. The search terms were: “surgery for Crohn’s”, “bowel surgery for Crohn’s”, “bowel resection for Crohn’s”, “complications of Crohn’s surgery”, “risks of Crohn’s surgery” and “recovery following Crohn’s surgery”. The search was run again in March 2025 before preparing this manuscript to ensure that the results had not changed.

Searches took place using the ‘incognito’ setting on Google, creating a private window without personalisation. This prevents the search results being influenced by previous searches that are tailored to the reviewer’s history. Only the results from the first two pages for each search term were reviewed to reflect that 99% of internet users will only access information from the first page of results.^22^

The results of each search were screened against the eligibility criteria and duplicates were removed. Searches and screening were performed by AW. Eligibility of shortlisted websites was double-checked by JJ, and disagreements were resolved through discussion or by a third reviewer (NH).

### Eligibility criteria

To be included in the review, the websites had to contain information on bowel resection to treat CD and be aimed at lay patient readers. Only sources written in the English language were included. Academic sources not aimed at lay readers were excluded, as were healthcare operator advertisements (because of the risk of biased information).^23^ Social media pages and newspaper articles were excluded because these often represent opinion rather than impartial information. This does not include medically reviewed online health articles which were still included in the study. Sources that only discussed medical management or surgeries other than bowel resection were excluded.

### Data collection

Data were collected independently by two researchers (AW and JJ), with any differences resolved by a third reviewer (NH). Data were extracted and entered into a form created on a Google Sheet (Google LLC) about:

• website descriptors
• description of CD
• treatment options
• description of bowel resection

Data were also gathered on whether surgery for ileocolic CD was mentioned as an alternative to medical therapy in the light of current evidence supporting earlier surgery.

A complete list of all the fields in the data extraction form is supplied in Supplementary File S1.

### Statistical analysis

The quality of a source was assessed using the DISCERN tool. This is a validated 16-item questionnaire designed to assess the quality of written information on treatment options.^24^ For each item, a score of 1 indicates low quality, and a score of 5 indicates excellent quality.

Readability was assessed using the widely used Flesch–Kincaid (FK) readability ease tool.^25,26^ This evaluates the average sentence length and syllables per word of a diverse range of publishing formats.^27^ An overall score of 0–100 is given. The higher the score, the easier the text is to read and understand. Each score equates to a US school grade. The complete tools are provided in Supplementary File S1.

We did not originally aim to assess the accessibility adjustments of websites. During the initial screening, it became apparent that websites differed significantly in terms of how accessible they are. A decision was therefore made to also analyse this aspect. The Web Content Accessibility Guidelines (WCAG) provide an internationally recognised set of recommendations for improving website accessibility.^28^ Accessibility adjustments described by WCAG to improve accessibility – such as adjustable text, transcripts for videos, screen reader (text to speech), alternative background colour and compatibility with external assistive technologies – were assessed.^29^

Given that use of artificial intelligence (AI) as part of online searches is increasingly popular, we comment on the AI-generated summary for each search term on Google. This summary appears at the top of the results page and the websites used to generate it are referenced. We explored the influence of websites included in our review on the AI-generated summaries.

## Results

**Figure 1:**
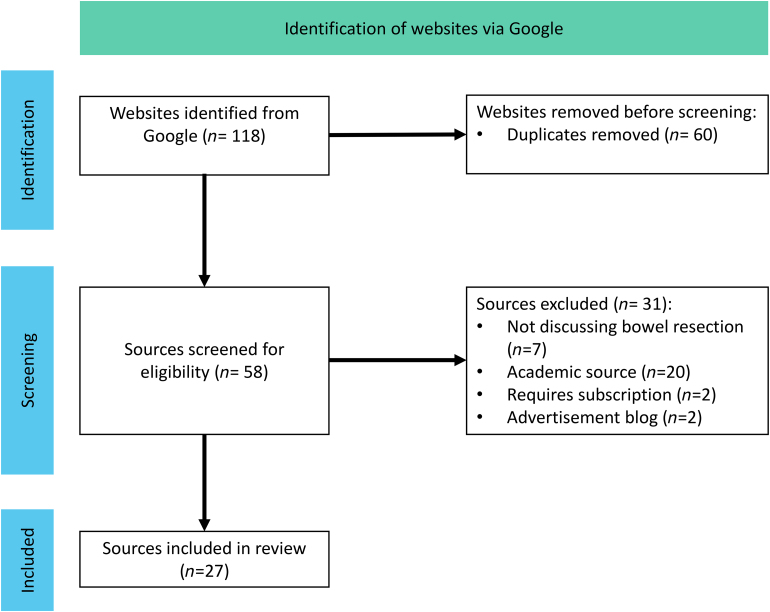
PRISMA diagram

The search, using the 6 search terms, yielded a total of 118 websites (from the first 2 results pages). Sixty were duplicates and were removed. The remaining 58 were screened for eligibility. Thirty-one sources were excluded with reasons, leaving twenty-seven sources available for analysis in the review. The results were consistent in our updated search in March 2025. The PRISMA diagram (Figure 1) summarises the screening process.

### Website descriptors

Most of the websites originated from the USA (*n* = 18), with the remainder from the UK (*n* = 5), Canada (*n* = 2) and Australia (*n* = 2). The most common publishing source was online medically reviewed health articles (*n* = 9), with other sources being hospital/specialty associations (*n* = 7), charities (*n* = 5) and healthcare resources (*n* = 4). Only five websites incorporated accessibility adjustments, with the most common being adjustable font (*n* = 5) and seizure safe content (*n* = 3). The characteristics of the websites and accessibility adjustments are included in Table 1.

**Table 1:** Website descriptors

Source	Title of website	Format of website	Name of uploader	Country of origin	Upload source type	Surgical procedure discussed	Accessibility adjustments	Examples of adjustments
https://crohnsandcolitis.org.uk/info-support/information-about-crohns-and-colitis/all-information-about-crohns-and-colitis/surgery-and-complications/surgery-for-crohns-disease	Crohn’s & Colitis UK	Html	Crohn’s & Colitis UK	UK	Charity	Stricturoplasty, resection, ileocecectomy, colectomy, proctocolectomy, ileostomy	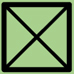	Adjustable text (font, size and line spacing), background colour, video transcripts, screen reader, auditory recording, seizure safe, compatible with assistive technologies
https://crohnsandcolitis.ca/About-Crohn-s-Colitis/IBD-Journey/Treatment-and-Medications/Surgery	Crohn’s & Colitis Canada: Surgery	Html	Crohn’s & Colitis Canada	Canada	Charity	Proctocolectomy, ileoanal pouch anastomosis, stricuroplasty, resection	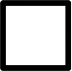	
https://www.nhs.uk/conditions/crohns-disease/treatment/	NHS: Treatment for Crohn’s disease	Html	NHS	UK	Healthcare	Resection, ileostomy	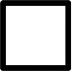	
https://www.webmd.com/ibd-crohns-disease/crohns-disease/surgery-for-crohns-disease	WebMD: Surgery for Crohn’s disease	Html	WebMD	USA	Other (online health article)	Stricturoplasty, fistulotomy, colectomy, proctocolectomy, bowel resection, ileostomy	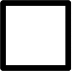	
https://www.healthdirect.gov.au/surgery/abdominal-surgery-for-crohns-disease	HealthDirect Australia: Abdominal surgery for Crohn’s disease	Html	HealthDirect Australia	Australia	Healthcare	Resection with colostomy/ ileostomy	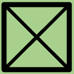	Adjustable text (size, font and line spacing), background colour, auditory recording
https://www.healthline.com/health/crohns-disease/surgery	Healthline Media: Surgery for Crohn’s disease	Html	Healthline Media	USA	Other (online health article)	Ileostomy, colostomy, proctocolectomy, bowel resection, stricturoplasty	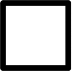	
https://www.everydayhealth.com/hs/crohns-disease-treatment-management/crohns-surgery-right-for-you/	Everyday Health: Should i get surgery for Crohn’s disease?	Html	Everyday Health	USA	Other (online health article)	Stricturoplasty, fistulotomy, bowel resection, colectomy, proctocolectomy	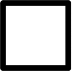	
https://nyulangone.org/conditions/crohns-disease-in-children/treatments/surgery-for-crohns-disease-in-children	Hanssenfeld Children’s Hospital: Surgery for Crohn’s disease in children	Html	NYU Lagoon Health	USA	Hospital/specialty association	Small bowel resection, ileostomy, stricturoplasty, colectomy, proctocolectomy	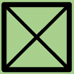	Adjustable text (size, font and line spacing), seizure safe
https://www.circlehealthgroup.co.uk/treatments/abdominal-surgery-for-crohns-disease	Abdominal surgery for Crohn’s disease	Html	Circle Health Group	UK	Hospital/specialty association	Bowel resection, stricturoplasty, colostomy, ileostomy	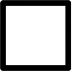	
https://generalsurgery.ucsf.edu/conditions–procedures/crohns-disease.aspx	UCSF general surgery: Crohn’s disease	Html	University of California San Francisco	USA	Hospital/specialty association	Small bowel resection, colectomy, proctocolectomy, ileostomy	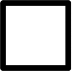	
https://www.verywellhealth.com/crohn-s-disease-surgery-overview-5206218	Crohn’s disease surgery: Overview	Html	Verywell Health	USA	Other (online health article)	Abscess drainage, colectomy, fistulotomy, ileostomy, proctocolectomy, bowel resection, stricturoplasty	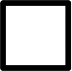	
https://www.medicalnewstoday.com/articles/323236	Crohn’s disease: What to know	Html	Medical News Today	USA	Other (online health article)	Small bowel resection, subtotal colectomy, stricturoplasty, proctocolectomy, ileostomy	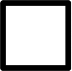	
https://www.crohnscolitisfoundation.org/what-is-crohns-disease/treatment/surgery/small-large-bowel-resection	Crohn’s & Colitis Foundation: Small and large bowel resection	Html	Crohn’s & Colitis Foundation	UK	Charity	Small bowel resection, large bowel resection	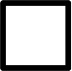	
https://www.webmd.com/ibd-crohns-disease/crohns-disease/resection-surgery-crohns-disease	WebMD: Resection for Crohn’s disease	Html	WebMD	USA	Other (online health article)	Small, large and ileocecal resection	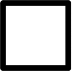	
https://www.medicalnewstoday.com/articles/324302	Bowel resection for Crohn’s disease: What to expect	Html	Medical News Today	USA	Other (online health article)	Small bowel resection, structuroplasty, colectomy, proctocolectomy	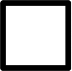	
https://www.healthcentral.com/condition/crohns-disease/bowel-resection	How bowel resection surgery is used for Crohn’s disease	Html	HealthCentral	USA	Other (online health article)	Small bowel resection, ileocecal resection, large bowel resection, colectomy, stricturoplasty	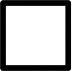	
https://www.everydayhealth.com/crohns-disease/treatment/tips-successful-recovery-from-crohns-surgery/	Everything you need to know about Crohn’s disease surgery	Html	Everyday health	USA	Other (online health article)	Bowel resection	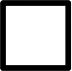	
https://nyulangone.org/conditions/inflammatory-bowel-disease/treatments/surgery-for-inflammatory-bowel-disease	Surgery for inflammatory bowel disease	Html	NYU Lagoon Health	USA	Hospital/specialty association	Small bowel resection, structuroplasty, colectomy, proctocolectomy, ileostomy	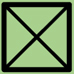	Adjustable text (size, font and line spacing), seizure safe
https://www.ibdrelief.com/learn/treatment/surgery/small-intestine-resection-surgery-for-ibd	Small intestine resection surgery for IBD	html	IBD Relief	UK	Charity	Small bowel resection	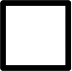	
https://crohnsandcolitis.org.au/living-with-crohns-colitis/medical/complications-of-ibd/	Crohn’s & Colitis Australia: Surgery	Html	Crohn’s & Colitis Australia	Australia	Charity	Ileocecal resection, structuroplasty, colectomy, ileostomy, ileal pouch–anal anastomosis, proctocolectomy	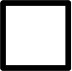	
https://www.medicinenet.com/can_crohns_disease_be_cured_with_surgery/article.htm	MedicineNet: Can Crohn’s disease be cured with surgery?	Html	Dr Jasmine Shaikh, MD	USA	Healthcare professional	Stricturoplasty, fistulotomy, colectomy, proctocolectomy, ileostomy, bowel resection	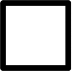	
https://www.verywellhealth.com/resection-surgery-for-crohns-disease-1943058	Resection surgery for Crohn’s disease	Html	Verywell Health	USA	Other (online health article)	Bowel resection	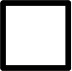	
https://myhealth.alberta.ca/Health/aftercareinformation/pages/conditions.aspx?hwid = zc2026	Laparoscopic bowel resection: What to expect at home	Html	MyHealth Alberta	Canada	Hospital/specialty association	Laparoscopic bowel resection (recovery)	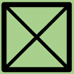	Adjustable text (font style)
https://www.niddk.nih.gov/health-information/digestive-diseases/crohns-disease/treatment	National Institute of Diabetes and Digestive and Kidney disease: Treatment for Crohn’s disease	Html	National Institute of Diabetes and Digestive and Kidney Disease	USA	Healthcare	Small bowel resection, subtotal colectomy, proctocolectomy and ileostomy	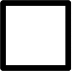	
https://www.healthcentral.com/condition/crohns-disease/crohns-disease-surgery	Crohn’s disease surgery: Ostomy, ileostomy, stricturoplasty and more	Html	Health Central	USA	Other (online health article)	Stricturoplasty, fistulotomy, colectomy, proctocolectomy, small/large bowel resection	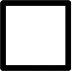	
https://my.clevelandclinic.org/health/treatments/24596-small-bowel-resection	Small bowel resection surgery: Procedure and purpose	Html	Cleveland clinic	USA	Hospital/ specialty association	Small bowel resection	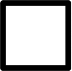	
https://www.mayoclinic.org/diseases-conditions/crohns-disease/diagnosis-treatment/drc-20353309	Crohn’s disease: Diagnosis and treatment	Html	Mayo clinic	USA	Hospital/specialty association	Bowel resection, fistula, abscess	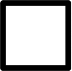	

NHS = National Health Service.

### Health condition

All sources discussed surgery for CD. Fewer than half of the sources discussed the signs and symptoms (*n* = 12) and only 8 discussed medical therapy. Few described the causes (*n* = 4) and classification of phenotype (*n* = 4). A summary of aspects of CD discussed on the websites is provided in Supplementary File S2.

### Bowel resection

More than half of the sources discussed the different types of bowel resection (*n* = 17), with small bowel resection the most frequently mentioned (*n* = 17), followed by large bowel resection (*n* = 9) and ileocolic resection (*n* = 8). Only one source, Crohn’s & Colitis UK (CCUK), described ileocolic resection as an alternative option to medical therapy in the management of ileocolic CD. The most common topics discussed relating to bowel resection were indications for surgery (*n* = 24) and postoperative recovery (*n* = 22). Nineteen sources mentioned the risks of bowel resection, and eighteen highlighted the benefits. Fewer than half discussed the risk of recurrence postoperatively (*n* = 15) and only six highlighted the postoperative management of CD. Lifestyle changes post-resection were referenced in 11 of the sources. Four websites had incorrect data (examples provided in Supplementary File S2). The various aspects of surgery described on the websites are summarised in Table 2.

**Table 2:** Descriptions of bowel resection for Crohn’s disease

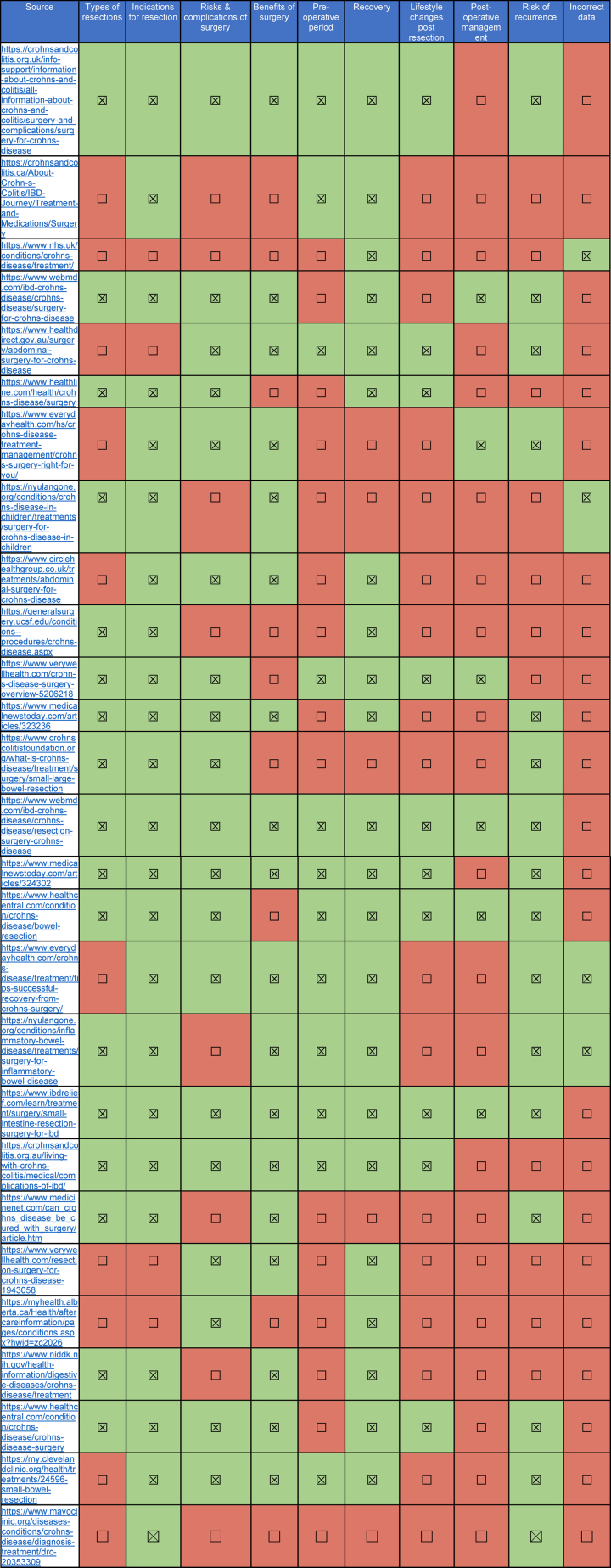

### AI-generated summaries

Supplementary File S2 shows a list of websites used to generate the AI-powered summaries for each search term: 51.5% of these references are patient-focused websites included in our review.

### DISCERN

The mean DISCERN score of the online sources was 3.1 out of 5 (standard deviation [sd] ±0.80), which equates to moderate quality. No source had a score of 1, twelve had a score of 3 and one source had a score of 5 (indicating excellent quality).

Ten sources scored 4 or more (good to excellent quality), scoring highly in the domains of transparency of where and when information was sourced from and providing additional sources of support. By contrast, websites that were of ‘low’ and ‘moderate’ quality (*n* = 16) lacked transparency and focused more on the benefits of treatment options while neglecting the risks.

With the exception of the CCUK website, all the sources scored between 1 and 3 in domain 13 (description of the impact of treatment on quality of life). Eleven of the sources scored either low or low–moderate on domain 15 (support for SDM). Table 3 shows the individual DISCERN score for each website. Supplementary File S2 provides the overall DISCERN score along with the overall quality rating.

**Table 3:** DISCERN assessment

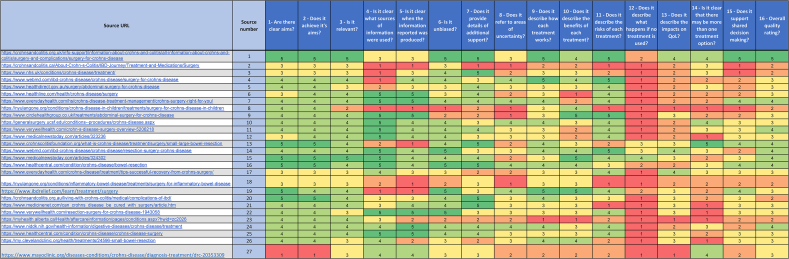

Overall quality rating 1 = poor; 2 = poor to moderate; 3 = moderate; 4 = good; 5 = excellent

### Flesch–Kincaid

The mean FK score was 51.9 (sd ±8.70), which represents the reader needing to reach US Grade 10 to 12 or UK A-level standard to understand the text. The FK scores are shown in Table 4, along with the UK school year that the reader would need to reach to fully understand the text.

**Table 4:** Flesch–Kincaid assessment

Website number	Website URL	Flesch–Kincaid score	UK school year
1	https://crohnsandcolitis.org.uk/info-support/information-about-crohns-and-colitis/all-information-about-crohns-and-colitis/surgery-and-complications/surgery-for-crohns-disease	67.5	9–10
2	https://crohnsandcolitis.ca/About-Crohn-s-Colitis/IBD-Journey/Treatment-and-Medications/Surgery	50.1	11–13
3	https://www.nhs.uk/conditions/crohns-disease/treatment/	52.6	11–13
4	https://www.webmd.com/ibd-crohns-disease/crohns-disease/surgery-for-crohns-disease	59.8	11–13
5	https://www.healthdirect.gov.au/surgery/abdominal-surgery-for-crohns-disease	54.1	11–13
6	https://www.healthline.com/health/crohns-disease/surgery	44.6	University
7	https://www.everydayhealth.com/hs/crohns-disease-treatment-management/crohns-surgery-right-for-you/	46.2	University
8	https://nyulangone.org/conditions/crohns-disease-in-children/treatments/surgery-for-crohns-disease-in-children	37.3	University
9	https://www.circlehealthgroup.co.uk/treatments/abdominal-surgery-for-crohns-disease	58.6	11–13
10	https://generalsurgery.ucsf.edu/conditions–procedures/crohns-disease.aspx	53.1	11–13
11	https://www.verywellhealth.com/crohn-s-disease-surgery-overview-5206218	50.4	11–13
12	https://www.medicalnewstoday.com/articles/323236	43.4	University
13	https://www.crohnscolitisfoundation.org/what-is-crohns-disease/treatment/surgery/small-large-bowel-resection	50	11–13
14	https://www.webmd.com/ibd-crohns-disease/crohns-disease/resection-surgery-crohns-disease	60.8	9–10
15	https://www.medicalnewstoday.com/articles/324302	42.2	University
16	https://www.healthcentral.com/condition/crohns-disease/bowel-resection	47.6	University
17	https://www.everydayhealth.com/crohns-disease/treatment/tips-successful-recovery-from-crohns-surgery/	48.58	University
18	https://nyulangone.org/conditions/inflammatory-bowel-disease/treatments/surgery-for-inflammatory-bowel-disease	37.4	University
19	https://www.ibdrelief.com/learn/treatment/surgery/small-intestine-resection-surgery-for-ibd	46.9	University
20	https://crohnsandcolitis.org.au/living-with-crohns-colitis/medical/complications-of-ibd/	47	University
21	https://www.medicinenet.com/can_crohns_disease_be_cured_with_surgery/article.htm	31.3	11–13
22	https://www.verywellhealth.com/resection-surgery-for-crohns-disease-1943058	49.3	11–13
23	https://myhealth.alberta.ca/Health/aftercareinformation/pages/conditions.aspx?hwid = zc2026	77.8	8
24	https://www.niddk.nih.gov/health-information/digestive-diseases/crohns-disease/treatment	44.6	University
25	https://www.healthcentral.com/condition/crohns-disease/crohns-disease-surgery	49.7	University
26	https://my.clevelandclinic.org/health/treatments/24596-small-bowel-resection	44.3	University
27	https://www.mayoclinic.org/diseases-conditions/crohns-disease/diagnosis-treatment/drc-20353309	51.9	11–13

Overall, there were few disagreements between the two reviewers (AW and JJ) relating to the overall DISCERN score (*n* = 5) and FK grading (*n* = 4). These were all resolved by a third reviewer (NH).

## Discussion

This study systematically reviewed the web-based patient information on bowel resection for CD. Of the 57 sources identified from initial searches (after the removal of duplicates), fewer than half were targeted to patients while also discussing bowel resection as a treatment option. Patients who are considering a bowel resection are therefore restricted to a limited number of patient-focused online sources.

The different types of bowel resection were only discussed in 17 of the 27 included websites. With CD having a varied phenotype, the type of bowel resection required to appropriately manage the disease will vary from patient to patient. This could range from a limited ileal resection to an extended colectomy. Each type of resection will have different implications for the patient’s recovery, lifestyle and the risk of adverse outcomes, including morbidity rates. Consequently, generic information on ‘bowel resection’ is unlikely to be sufficient to inform patients on the risks and benefits of the specific resection they are considering. Despite ileocolic resection being the most frequently performed procedure in the treatment of terminal ileal CD, it was only discussed in a handful of sources (*n* = 8).^30^ With the exception of CCUK, none of the websites described surgery as an alternative option to medical therapy, despite accumulating evidence in favour of ‘earlier’ surgery. Instead, the continuing theme of surgery as an option when medical therapy ‘fails’ was projected onto patients.

The mean DISCERN score of 3.1 (indicating websites of moderate quality) suggests that online information on bowel resection for CD is currently of inadequate quality for patient readers. Nearly half of the websites assessed were deemed poor at supporting SDM. This is disappointing in the light of accumulating evidence that patients’ involvement in their care is a strong determinant of patient satisfaction.^31^ Because more than half of the sources available fail to discuss both the advantages and disadvantages of each treatment option, even patients who proactively perform their own research online may be unable to fully engage with their healthcare professionals in discussions around treatment options.

The mean FK score of 51.9 means that readers require A-level (UK), Grade 12 (USA) or European Baccalaureate (or equivalent) level of education to fully understand the text. Key organisations have recommended that patient education materials should be written at or below a sixth grade reading level, roughly corresponding to that of an 11-year-old.^32–34^ With only three sources meeting this recommended standard, our review highlights that the online patient information is not written in appropriate lay language for patient readers. The incidence of CD is rising amongst the younger age groups, which makes this finding significant.^35,36^

Our review identified only two websites that were both of high quality in terms of information provided and easy to read: the CCUK website (a charity organisation) and a medically reviewed online health article produced by WebMD. This is a positive finding, because many patients are directed to CCUK by clinicians in the UK for information regarding CD. Conversely, the Crohn’s & Colitis Canada and Crohn’s and Colitis Foundation websites (Canadian and American equivalent, respectively) would require a higher level of education to be understood based on their FK scores. Surprisingly, the National Health Service (NHS) website was not one of these highly rated sources, despite being trusted by many patients in the UK as a source of reliable and creditable online health information.

More than half of health-related searches generated AI summaries according to an article published in 2024.^37^ It is likely that this percentage has since increased. The extent to which a patient relies on these summaries for information is not known. However, given these summaries appear at the top of a results page, it is likely that patients read them first. We found that the summaries are predominantly produced using information from the patient-focused websites assessed in this review. Therefore, it is crucial that the information on these websites is of adequate quality. Assessing the accuracy of these summaries was not within the scope of this review but would be valuable to explore in the future.

With one in six people living with an impairment or disability globally, key organisations are recognising the importance of making information understandable and have provided recommendations on making websites more accessible.^38–40^ Only one in five websites in this review incorporated these adjustments. This is clearly an area in which website creators need to improve. CCUK provides the highest number of accessibility adjustments among websites in this review, including for those with visual impairments, learning difficulties (such as dyslexia) and seizure, allowing their site to be more inclusive.

Our study has several strengths. Search terms were generated by patient experts. Screening, data extraction and analysis was done by two reviewers, which increases the confidence in the validity of the scores. This ensures that websites that patients commonly access are included in this systematic review. We also included an assessment of accessibility adjustment, which has not been considered in other studies assessing the quality of online patient information (relating to other conditions).^41–43^

### Study limitations

This study does have its limitations. Online health videos were excluded but are increasingly being used by patients to seek support and access health-related information.^44^ The DISCERN score, although trusted by many studies to assess quality, still has limitations. The reliability of the DISCERN results improves with training because the 16-item questionnaire contains both subjective and objective questions. This means that the scores are likely to vary between reviewers. As described above, however, in our study, there was high level of agreement between the scores from AW and JJ. Finally, the searches were performed in the UK; the results may be different if the searches are performed in another country. Newly diagnosed patients are also likely to access social media platforms as an alternative source of information. Inclusion of social media could have altered our findings and would be valuable to explore in the future.

As a way of improving online patient information, website creators should consider working alongside patients, professional bodies and clinicians to design websites. Clinicians in the UK commonly direct their patients to CCUK for further reading. Because it scored well across all the areas assessed in this review, clinicians should continue to direct their patients to this website. Key areas for the improvement of websites that performed less well in this review are: their transparency in terms of where and when the online information was collected; recommendations to support SDM among patients; and the comprehensiveness of discussions on benefits and risks of treatment options to allow patients to make informed choices.

## Conclusion

The findings of this systematic review highlighted the limitations of the current online patient information surrounding bowel resection in CD. The involvement of patients, working alongside professional bodies and clinicians, in the development of health-related websites is recommended.

## Competing interests

The author/s declare no competing interests.

## Funding

The author/s received no financial support for the research, authorship and/or publication of this article.

## Ethics approval and consent to participate

Not applicable.

## Author contributions

All authors contributed to the study design. AW, JJ and NH collected data for the study. AW wrote the first draft of the paper. All authors revised the article for important intellectual content, provided final approval of the version to be published, and agreed to be accountable for all aspects of the work.

## Artificial Intelligence

The author/s declare that no AI was used to conduct the study or prepare the manuscript.

## Supplementary Information

The online version contains supplementary material available at https://doi.org/10.1308/rcsann.2025.0108.
